# Bacteriophage Therapy: Clinical Trials and Regulatory Hurdles

**DOI:** 10.3389/fcimb.2018.00376

**Published:** 2018-10-23

**Authors:** Lucy L. Furfaro, Matthew S. Payne, Barbara J. Chang

**Affiliations:** ^1^Division of Obstetrics and Gynecology, School of Medicine, The University of Western Australia, Crawley, WA, Australia; ^2^The Marshall Centre for Infectious Diseases Research and Training, School of Biomedical Sciences, The University of Western Australia, Crawley, WA, Australia

**Keywords:** bacteriophage, phage therapy, regulations, clinical trials, antimicrobial resistance, alternative treatments

## Abstract

Increasing reports of antimicrobial resistance and limited new antibiotic discoveries and development have fuelled innovation in other research fields and led to a revitalization of bacteriophage (phage) studies in the Western world. Phage therapy mainly utilizes obligately lytic phages to kill their respective bacterial hosts, while leaving human cells intact and reducing the broader impact on commensal bacteria that often results from antibiotic use. Phage therapy is rapidly evolving and has resulted in cases of life-saving therapeutic use and multiple clinical trials. However, one of the biggest challenges this antibiotic alternative faces relates to regulations and policy surrounding clinical use and implementation beyond compassionate cases. This review discusses the multi-drug resistant Gram-negative pathogens of highest critical priority and summarizes the current state-of-the-art in phage therapy targeting these organisms. It also examines phage therapy in humans in general and the approaches different countries have taken to introduce it into clinical practice and policy. We aim to highlight the rapidly advancing field of phage therapy and the challenges that lie ahead as the world shifts away from complete reliance on antibiotics.

## The challenge of multi-drug resistant bacteria

In 2017 the World Health Organization published a list of global priority pathogens comprising 12 species of bacteria categorized into critical, high and medium priority based on their level of resistance and available therapeutics (Tacconelli et al., 2018). The current rate of resistance development far exceeds the level of antibiotic discovery and development and represents a global public health challenge. Estimates have suggested that upwards of 10 million people could die each year due to antimicrobial resistance by 2050 (O'neill, 2014). While this is a contentious figure (De Kraker et al., 2016), it nonetheless highlights the serious problem we face regarding therapeutic options for multi-drug resistant (MDR) bacterial infections (Bassetti et al., 2017). The natural predators of bacteria are the bacterial viruses known as bacteriophages or phages. Found ubiquitously, these organisms are estimated to be present at numbers equivalent to a trillion per grain of sand on Earth (Keen, 2015). Evolving in parallel with bacteria, phages are potential antibacterial therapeutic agents against such MDR pathogens (Burrowes et al., 2011). Here we focus on three critical priority pathogens, *Acinetobacter baumannii, Pseudomonas aeruginosa*, and members of the *Enterobacteriaceae* (Tacconelli et al., 2018) and the current advances in phage therapy research to target these organisms, as well as exploring more general issues of clinical trials and regulatory complexities of phage therapy.

### Acinetobacter baumannii

*A. baumannii* is recognized as a critical priority pathogen due to the increasing incidence of antimicrobial resistance and significant role in nosocomial infections (Mcconnell et al., 2013). Around 20 years after an early trial of anti-*A. baumannii* phage therapy in mice (Soothill, 1992), a surge in reports of *A. baumannii* lytic phage isolation and their *in vitro* activity occurred, as reviewed by Garcia-Quintanilla et al. (2013). Since this time, significant advances have been made with further *in vitro* studies (Liu et al., 2016; Ghajavand et al., 2017) and numerous *in vivo* animal studies (Kusradze et al., 2016; Regeimbal et al., 2016; Yin et al., 2017; Zhou et al., 2018). Phage therapy evaluation in a mouse model of *A. baumannii* infection resulted in 2.3-fold increased survival in the phage-treated group compared to control groups (Cha et al., 2018).

A novel lysin from *A. baumannii* prophages with the capacity to kill clinical MDR isolates and rescue mice from lethal infections has also been characterized (Lood et al., 2015). The use of these enzymatic compounds is not a new concept; and although lysin use has been restricted in Gram-negative bacteria due to their outer membrane barrier, a rise in the literature suggests that this no longer poses a constraint on lysin use in Gram-negatives (Thandar et al., 2016; Peng et al., 2017; Larpin et al., 2018).

Advances have also been made in human phage therapy trials. A key case in the United States involved the first intravenous administration of phage therapy and resulted in the successful treatment and recovery of a patient with *A. baumannii* pancreatic pseudocyst infection (Schooley et al., 2017). This has led to increased phage therapy exposure to the public and arguably increased clinical awareness regarding this alternative therapeutic. More recently another case study involving infection at a craniectomy site with a MDR-*A. baumannii*, applied a personalized phage cocktail intravenously in an attempt to improve patient outcomes (Lavergne et al., 2018). Unfortunately, the patient passed away after life support efforts were ceased following the family's request. In cases such as this it is difficult to navigate the regulatory issues in a timely manner; while personalized therapy is ideal to adapt to patient needs it can be a challenge.

### Pseudomonas aeruginosa

*P. aeruginosa* is a major opportunistic pathogen and cause of nosocomial infections (Lyczak et al., 2000; Breidenstein et al., 2011). It is also a frequent cause of chronic lung infections in cystic fibrosis patients and as such has been assessed as a target for phage therapy (Olszak et al., 2015). Phage therapy for *P. aeruginosa* infections dates back more than 50 years (Bertoye et al., 1959; Soothill, 2013), but recent developments in use of both phage lysins and live phage are very promising. A review by Rossitto and colleagues describes the current literature in this field and the challenges associated with phage therapy in cystic fibrosis, in particular they suggest that future studies include testing on both mucoid and non-mucoid *P. aeruginosa* isolates and the use of both pulmonary and non-pulmonary host models (Rossitto et al., 2018). Spray-dried formulations of phages have also been thoroughly tested for inhaled application against *P. aeruginosa* lung infection (Chang et al., 2017, 2018). Immunogenicity data has been assessed using an *in vitro* human lung model and demonstrated an increase in IL-6 and TNF-a for one of two phages (Shiley et al., 2017). The human immune response is an important consideration when assessing therapeutic phage application (Krut and Bekeredjian-Ding, 2018) however, beneficial effects of the immune response conducive to positive phage therapy outcomes have also been reported (Roach et al., 2017).

A cocktail of six phages was observed to successfully treat respiratory *P. aeruginosa* infection in mice and, additionally, sepsis in *Galleria mellonella* models (Forti et al., 2018). Ability of some phages to penetrate *P. aeruginosa* biofilms is another major advantage over conventional treatments (Fong et al., 2017; Waters et al., 2017), while co-administration of phages and antibiotics has been reported as a mechanism of restoring antibiotic sensitivity (Chan et al., 2016). In the latter case, where phages utilize components of multidrug efflux pump systems as receptors, mutation to confer phage-resistance alters the pump mechanism, leading to antibiotic re-sensitization. A case study of aortic prosthetic graft infection by *P. aeruginosa* with direct administration to the graft of a combination of phage and ceftazidime was successful in resolving and possibly eradicating infection (Chan et al., 2018). Finally, phage lysin research is also on the increase: Guo et al. described a novel endolysin with *in vitro* activity against *P. aeruginosa* and other Gram-negative bacteria on the critical priority pathogens list (Guo et al., 2017), with similar reports from other groups (Larpin et al., 2018).

### Enterobacteriaceae

Within the family *Enterobacteriaceae, Escherichia coli*, and *Klebsiella* spp. ranked highest in the WHO critical priority list of antibiotic-resistant bacteria followed by *Enterobacter, Serratia* and *Proteus* spp. (Tacconelli et al., 2018). While occupying many commensal niches, *E. coli* isolates include significant intestinal and extraintestinal pathogens (Bolocan et al., 2016). The majority of early phage research was undertaken with coliphages (phages that infect *E. coli*), particularly T4 (Stahl, 1989; Edgar, 2004), evidenced by numerous studies, some of which are summarized by Bolocan et al. (2016). More recently, *in vitro* and *in vivo* studies have shown promising results, for example control of enteropathogenic *E. coli* in mice with hospital sewage-isolated phage (Vahedi et al., 2018) and effect of coliphages against planktonic and biofilm-associated infections (Tkhilaishvili et al., 2018).

*Klebsiella* spp. are frequent nosocomial and community-acquired pathogens recognized for their MDR status. Cao et al. administered intranasal phage to treat *K. pneumoniae* lung infection in mice, resulting in protection against lethal infection and lower inflammatory cytokine levels in the lung (Cao et al., 2015). Similarly, in a burn wound mouse model of *K. pneumoniae* infection, topical phage application resulted in a significant reduction in mortality (Kumari et al., 2011) and a liposome loaded phage cocktail enhanced bacterial clearance and rate of healing (Chadha et al., 2017).

Other phage therapeutic uses include prevention of biofilm formation. Depolymerase producing *K. pneumoniae* phage, in combination with iron antagonizing agents, showed ability to eradicate early biofilms of *K. pneumoniae*: a promising preventative strategy (Chhibber et al., 2013). Progress has also been made in lysin research: Yan et al. described a novel fusion protein that combines the receptor binding domains of colicin A with an *E. coli* phage lysin to overcome the blocking effect of the Gram-negative outer membrane, with successful control of *E. coli* both *in vitro* and in a mouse model (Yan et al., 2017).

While many studies have addressed phage therapy *in vitro* and *in vivo*, there is much further work required for translation into humans. Case reports have been discussed, but lack the robust evidence of clinical trials.

## Phage therapy in humans

Phage use in Eastern Europe and the former Soviet Union has been widespread since their discovery; as a result therapeutic phage use is integrated within their health care systems. However, this potential therapy is only recently being investigated according to rigorous scientific standards (Kutter et al., 2010; Villarroel et al., 2017). Abedon has presented a list of key criteria that should be thoroughly considered and reported in phage therapy studies (Abedon, 2017). Information critical to the success of clinical trials includes the adequate characterization and selection of phages as well as of the subjects (humans) and the target bacteria. Additional data are also required such as formulations, dosing and efficacy, however, without the foundation of characterized and well-planned targets these are of no value. Detailed reporting would improve the quality of future research and enable replication and extension of previous studies. Another consideration is the choice of appropriate disease targets for phage therapy (Harper, 2018). For example, the species specificity characteristic of most phages is generally highly desirable in monomicrobial diseases, however, this specificity can be a major limitation in cases of polymicrobial infections unless, perhaps, the phage is administered in combination with a suitable antibiotic. Such considerations are imperative for patient safety in clinical trials, as removal of one pathogen and consequent overgrowth of a second could potentially have fatal consequences (Harper, 2018). On the other hand, it may be that broad-host-range phages are more common than is currently believed, due in part to biases in phage isolation methods (De Jonge et al., 2018): this disparity deserves much further research.

## Clinical trials involving phages

One of the current challenges of progressing phage therapy into the clinic is the lack of validated and adequately controlled clinical trials. Additional care should be taken in the planning and design of such trials as, while clinical trial design for phage therapy will naturally share many parallels with standard drug clinical trials, there are several factors that are unique to phages. These include pharmacological considerations such as the dosage (Payne and Jansen, 2003). As these are self-replicating viruses, their dose has the potential to exponentially increase upon reaching the bacteria of interest. This leads to another consideration of application: phages require direct contact with the bacteria and if distributed too broadly they will be less efficacious. Topical applications have been widely used to address this, however, as mentioned other methods have been used with success. When considering monotherapy or combination therapy approaches, phage cocktails offer broad spectrum activity and reduce the chances of resistance formation, however, it should be noted that combination therapy greatly increases the challenge of assessing inflammatory effects, potential for gene transfer and phage resistance development for all phages in a cocktail (Parracho et al., 2012).

Some have argued that exposure to bacteriophages occurs in humans every day and is evidence of their safety, however, in the context of clinical trials there are a number of considerations that should be addressed. The first of these relates to the sterility and purity of the phage preparation. It is imperative that products exclude toxins and bacterial debris to comply with good manufacturing practice or equivalent quality assurance standards. Parracho et al. described the quality parameters recommended for bacteriophage products from the point of phage identification through to manufacturing processes (Parracho et al., 2012). Secondly, concerns surrounding the potential for the onset of toxic shock as a result of the bactericidal effect of phages must also be addressed. While this has been reported to not be an issue (Speck and Smithyman, 2016) and this method of bacterial killing is shared by bactericidal antibiotics (Dufour et al., 2017), this is a necessary safety consideration prior to clinical trials.

**Figure 1:**
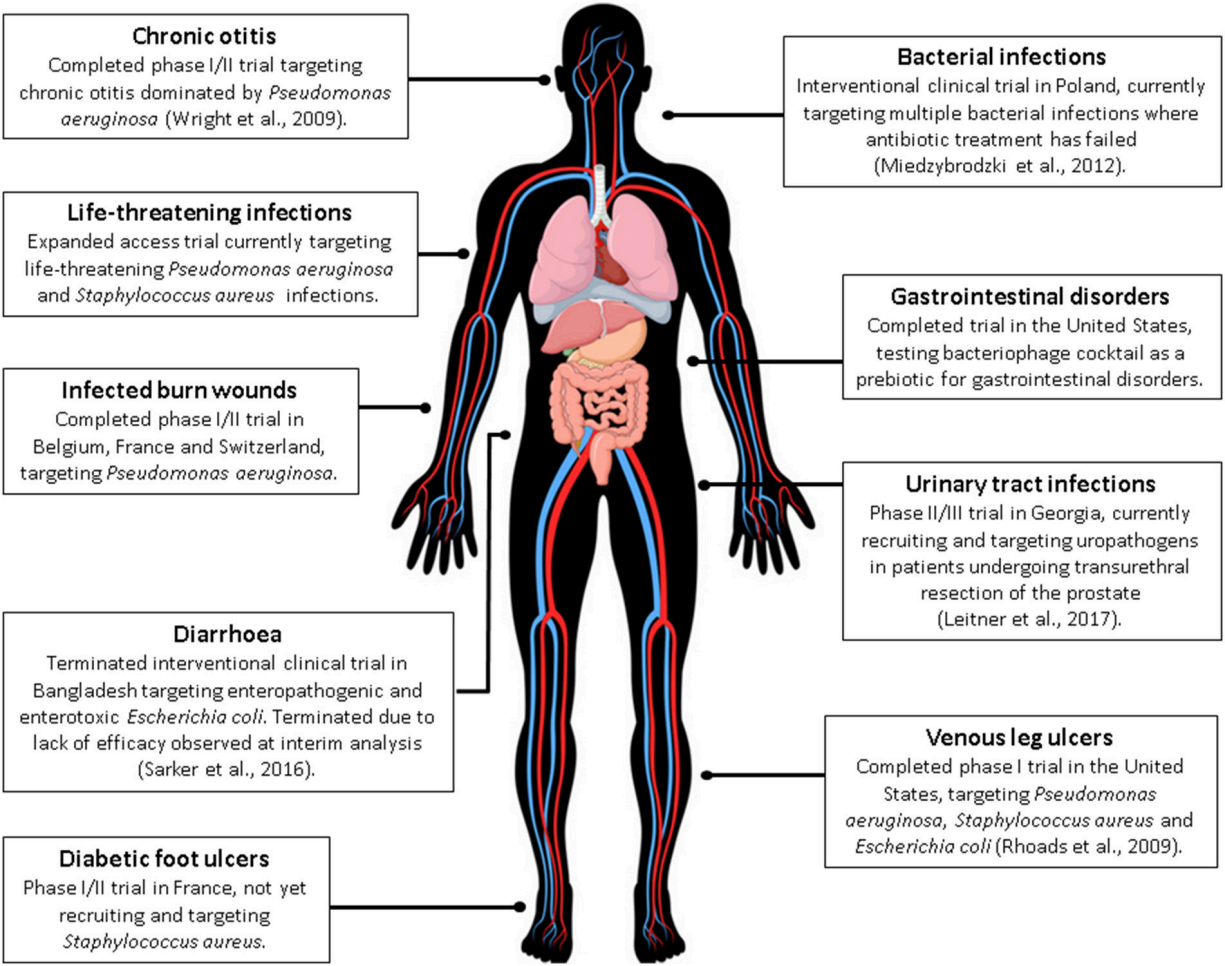
A current summary of human phage therapy trials and the range of target sites/infections (see www.clinicaltrials.gov or https://globalclinicaltrialdata.com/ where citation is not given). This figure includes a licensed image obtained by the authors.

Previous clinical trials involving phage therapy have been described in detail by Kutter et al. and include those undertaken in Georgia and Poland (Kutter et al., 2010). Worth noting are two phage therapy clinical trials that are used as examples throughout the literature, addressing safety of phages for treating venous leg ulcers (Rhoads et al., 2009) and safety and efficacy in chronic otitis (Wright et al., 2009). Rhoads and colleagues reported on safety in a small phase I trial in patients with venous leg ulcers and reported no adverse events with the administration of phages (Rhoads et al., 2009). Wright et al. demonstrated efficacy and safety of anti-Pseudomonal phages against late stage recurrent otitis which was dominated by MDR-*P. aeruginosa*. These are among the first controlled clinical trials in humans conducted in the western world. More recently, a number of clinical trials have been registered (https://clinicaltrials.gov/ and https://globalclinicaltrialdata.com/) as summarized in Figure 1 (Miedzybrodzki et al., 2012; Sarker et al., 2016; Leitner et al., 2017). At both web sites, use of the search phrase “phage therapy” resulted in 15 studies/trials in the former site, of which nine were phage therapy-related, with a focus on treatment of infection. Two additional studies were identified using the global clinical trials resource. Search results did not all represent standard clinical trials, for example sputum collections for *in vitro* phage testing and expanded access interventional trials were also included. Additional, scientifically sound clinical trials are vital to increasing the western clinical worlds' acceptance of phage therapy applications. While many observational studies have been conducted, these have been limited by small sample sizes and many are poorly controlled. Conversely, promising case studies do exist, however, robust clinical trial data is what is required by regulators in order to progress clinical guidelines for phage therapy.

## Regulation and policy development

No framework currently exists that explicitly defines phages in the context of medicinal products for use in humans, however, in Georgia these are embedded in the healthcare system as a standard medical application (Kutateladze, 2015). Specifically, the Eliava Institute of Bacteriophages, Microbiology and Virology has several phage preparations readily available (over-the-counter) and a broader range of products, specifically supplied to medical practitioners (Kutter et al., 2010; Kutateladze, 2015). Similarly, Poland has the Hirszfeld Institute of Immunology and Experimental Therapy, although this center supplies personalized phage products directly to physicians using a more tailored approach (Kutter et al., 2010). In other parts of the world, however, bacteriophages present a unique regulatory agenda.

Gorski summarizes the current access schemes around the world and identifies the main inclusion of compassionate use cases in most countries as a last resort option (Gorski et al., 2018). Schemes vary, however, all respond to the situation of a critically ill or chronically suffering patient for whom all authorized treatment options have been exhausted. While these schemes are beneficial in the short-term, it has been recognized that a dedicated phage therapy legal framework is essential for the smooth introduction of natural phage therapy into western medicine. Regulatory calls to action have been made in Europe with discussions around regulatory hurdles and future steps required to achieve appropriate phage-based therapeutic guidelines (Huys et al., 2013; Verbeken et al., 2014).

### Working towards a solution

A thorough analysis of key stakeholder opinions on the regulatory status of phage therapy was reported by Verbeken et al. (2014). Calls for two regulatory pathways were proposed, including product market placement of natural phage-based products and hospital exemption pathways for tailored phage therapy. The consensus among surveyed stakeholders was the need for a dedicated new regulatory framework for phage therapy and one which acknowledges the specific properties of phages and their interactions, in addition to the role of hospitals as providers of phage therapy (Verbeken et al., 2014). In the same vein, a workshop with the European Medicines Agency (EMA) set out to work together with all stakeholders to provide a solution to regulatory hurdles faced by phage researchers, while maintaining the standards of quality and safety (Pelfrene et al., 2016). Here, the EMA confirmed that none of the current regulations were suitable for phage therapy and discussed options for the way forward.

### Magistral phages

Political progression in Belgium has resulted in a magistral phage regulatory framework: a pragmatic framework to encompass tailored phage therapy (Pirnay et al., 2018). This regulatory framework includes a magistral formula in which non-authorized phage products can be prepared by a pharmacist, given the external quality assessment of the phage preparations. Quality assurance and good manufacturing practice are of extreme importance for any therapeutic agent and considerations for phage banks would include the characterization of all phages so that amongst other parameters, identity, viability, potency and purity are ensured (Pirnay et al., 2015; Pelfrene et al., 2016).

### A therapeutic classification for phages

Questions regarding the biological status of phages include whether they are living or not, which highlights the need for defined phage-specific terms of policy. As it currently stands, phage therapy in many cases represents the epitome of personalized medicine as it is a process involving tailor-made phage combinations specific for an individual patient's bacterial infection/s. This presents difficulties in the regulatory pipeline, as this move toward personalized medicine breaks the mold of regulatory conventions. It must be acknowledged that other therapeutics, for example cancer therapy (Daly, 2007), have faced a similar hurdle in the past and refinement is certainly possible. The Food and Drug Administration in the United States has recently provided an opportunity for the new Center for Innovative Phage Applications and Therapeutics (IPATH) to utilize phage therapy via the Emergency Investigational New Drug scheme. These initiatives are likely to improve clinical understanding and acceptance, while also providing supporting evidence of the need for dedicated regulatory guidelines.

## A future for phages

There is no doubt that phage therapy is an attractive solution to combating escalating antibiotic resistance. Numerous studies highlight the *in vitro* and *in vivo* potential of therapeutic phages and while a number of clinical trials have taken place over the last decade, further data is needed to present a robust regulatory case for clinical use. There remain obvious challenges ahead for phage therapy, particularly regarding management of regulatory policy. Progression toward novel schemes based around knowledge of phage applications should guide these processes and work toward a reasonable implementation structure. Ideally, regulatory developments should be reached in a standardized and global manner; however, this is understandably a challenge. While the field is rapidly progressing toward therapeutics, fuelled by the evident need for antibiotic alternatives, regulatory processes must be refined and approached from a novel phage-based perspective. One size does not fit all and collaborative efforts to build models that suit phages will surely result in better health outcomes for all. We must also remember that despite the frustrations of legislative parameters, it is of utmost importance to conserve high standards of safety, quality, and efficacy. It is vital that scientists and clinicians continue having these discussions with the appropriate regulatory bodies and move this area forward sooner rather than later.

## Author contributions

LF conceived the review topic and focus, drafted the manuscript and approved the final version to be published. Both BC and MP contributed to the structure and content, critically revised the drafted manuscript and approved the final version to be published.

### Conflict of interest statement

The authors declare that the research was conducted in the absence of any commercial or financial relationships that could be construed as a potential conflict of interest.
